# Strategies to Reduce Technique Failure in Peritoneal Dialysis

**DOI:** 10.34067/KID.0000000778

**Published:** 2025-04-24

**Authors:** Megha Salani

**Affiliations:** Vanderbilt University Medical Center, Nashville, Tennessee

**Keywords:** clinical epidemiology, dialysis, dialysis access, dialysis volume, ESKD, epidemiology and outcomes, peritoneal dialysis

The transition from peritoneal dialysis (PD) to hemodialysis is associated with significant morbidity and high health care costs.^1^ Previous studies evaluating the discontinuation of PD have used varying definitions of technique failure that sometimes include transplantation and death.^2^ By focusing only on transition to hemodialysis, we can identify more specific risk factors and postulate potential interventions to maintain patients on PD.

Technique failure can occur for a variety of reasons, including infection, mechanical problems, inadequate clearance or fluid removal, and psychosocial reasons.^1,2^ Whether the transition to hemodialysis is temporary or permanent is also variably defined in studies and can be difficult to discern in large retrospective studies.^2^ Both temporary and permanent transitions can result in hospitalizations and the need for hemodialysis catheters, which are associated with increased mortality and infection risk.^1^ Modality transitions are also times of psychologic stress for patients. In situations where health care providers are focused on life-saving interventions including modality change, patients often suffer from unaddressed anxieties and fears.^3^ Compared with PD, in-center dialysis has higher costs and a higher water footprint.^4,5^ All of these considerations warrant efforts to evaluate intervenable risk factors for technique failure.

In this issue of *Kidney360*, Adoukonou *et al.* present their retrospective analysis of data from the French Language Peritoneal Dialysis Registry (RDPLF) to evaluate risks factors for transition from PD to hemodialysis.^6^ The large cohort size of almost 16,000 patients and analysis over 12 years are strengths of the study. The most common cause of kidney failure in this cohort was vascular nephropathy, almost half of the patients had nurse-assisted care, and about 62% of patients in the study were on automated PD. These patient characteristics may not be generalizable to other regions. Approximately three-quarters of the patients were managed in small centers, which has been associated with a higher risk for technique failure in other studies.^7^

Data from such registries have multiple confounding factors. For example, if a subgroup has a high mortality rate, they may seem to have a lower rate of technique failure. However, this is because individuals in the subgroup did not survive long enough to develop complications necessitating a modality change rather than because of any protective factor. Adoukonou *et al.* used both the Cox model and the Fine-Gray model to improve their analysis in the face of competing events. They have thoroughly outlined the limitations of the conclusions that can be drawn from this retrospective study. Their work does demonstrate interesting trends, notably:

1. Suboptimal starters had an increased risk of early technique failure for psychosocial reasons.

Suboptimal starter status was defined as being on hemodialysis for fewer than 30 days before starting PD. This assumes a need for PD in the setting of an unplanned dialysis initiation, such as patients lacking prior nephrology care or those with an acute decline in kidney function requiring urgent initiation. Patients in this group had a high risk of early technique failure, which aligns with the assessment of the RDPLF registry by Lobbedez *et al*.^8^ Adoukonou *et al.* found that the most common causes of technique failure in this group were psychosocial reasons. This is likely multifactorial. There may be a lack of education or a lack of comprehension of education in the setting of acute illness and/or uremia. Patients may be emotionally distraught at the time of initiation, leading them to feel overwhelmed by home dialysis and self-care. Patients may have little to no residual kidney function after an AKI and require full-dose PD, which could potentially feel more burdensome than starting with incremental PD and gradually increasing the prescription over time.^8^

2. Continuous ambulatory PD (CAPD) had a higher rate of early technique failure from mechanical factors, and automated PD (APD) had a higher rate of late technique failure due to adequacy, infection, and mechanical issues.

Previous studies have shown conflicting results for peritonitis rates in APD versus CAPD.^9^ There is speculation that the decreased need for catheter manipulation in APD is protective. There is also speculation that peritonitis is more severe in APD due to later detection (due to being asleep while the effluent is draining) and decreased effectiveness of intraperitoneal antibiotics with rapid exchanges. However, there is not definitive evidence that either modality increases the risk of peritonitis.^9^ The variability in outcomes may reflect other risk factors that contribute more than modality type.

Similarly, the difference in infection and mechanical findings seen in the RDPLF registry may reflect comorbidities unrelated to PD modality. However, this does highlight the importance of considering a change between PD modalities for patients at risk for technique failure. Transitioning from APD to CAPD can increase clearance in patients with low transport status of the peritoneal membrane. Conversely, transitioning from CAPD to APD can help patients who are struggling with reabsorption and volume overload. Patients who are overwhelmed with the burden of manual exchanges may benefit from switching to APD. Patients who are unable to sleep well on the cycler or dislike being tethered to it could opt for CAPD.

3. Nurse-assisted PD had lower rates of early technique failure due to infection and late technique failure due to inadequate solute clearance.

A nurse that is well trained in PD could improve retention on PD by providing care with sterile technique, consistently choosing appropriate dextrose solutions to manage volume status, and troubleshooting issues early when they arise. Nurse assistance reduces the burden of care for patients and their caregivers. In the United States, nurse-assisted PD is often prohibitively expensive. Because PD is associated with less cost overall, it is conceivable that health care systems would be more inclined to cover the cost of nurse care if further studies bolster the findings of reduced risk of technique failure. This could be temporary in certain high-risk circumstances, such as with suboptimal starters, following hospitalizations, or for patients who need respite. Another consideration would be an option for expanding the use of PD in nursing facilities or group homes with appropriate staffing.

In summary, the data from the RDPLF registry offer insights into risk factors for transition from PD to hemodialysis, the causes of technique failure with which they are associated, and a general timeline of vulnerability after starting PD. Importantly, the data presented should not deter providers from offering PD to individuals who seem to be at higher risk of technique failure. Rather, the information should be used to help with assessment of risk and development of efforts to overcome obstacles and keep patients at home. In some situations, a transition to hemodialysis may be needed or desired over continuing PD. Having an awareness of who is at risk can allow for earlier education and planning and can help to dispel fears and ease the transition process for patients.
